# Bowhead and beluga whale acoustic detections in the western Beaufort Sea 2008–2018

**DOI:** 10.1371/journal.pone.0253929

**Published:** 2021-06-28

**Authors:** Kathleen M. Stafford, John J. Citta, Stephen R. Okkonen, Jinlun Zhang

**Affiliations:** 1 Applied Physics Laboratory, University of Washington, Seattle, Washington, United States of America; 2 Alaska Department of Fish and Game, Fairbanks, Alaska, United States of America; 3 Institute of Marine Science, University of Alaska Fairbanks, Fairbanks, Alaska, United States of America; Wildlife Conservation Society Canada, CANADA

## Abstract

The Distributed Biological Observatory (DBO) was established to detect environmental changes in the Pacific Arctic by regular monitoring of biophysical responses in each of 8 DBO regions. Here we examine the occurrence of bowhead and beluga whale vocalizations in the western Beaufort Sea acquired by acoustic instruments deployed from September 2008-July 2014 and September 2016-October 2018 to examine inter-annual variability of these Arctic endemic species in DBO Region 6. Acoustic data were collected on an oceanographic mooring deployed in the Beaufort shelfbreak jet at ~71.4°N, 152.0°W. Spectrograms of acoustic data files were visually examined for the presence or absence of known signals of bowhead and beluga whales. Weekly averages of whale occurrence were compared with outputs of zooplankton, temperature and sea ice from the BIOMAS model to determine if any of these variables influenced whale occurrence. In addition, the dates of acoustic whale passage in the spring and fall were compared to annual sea ice melt-out and freeze-up dates to examine changes in phenology. Neither bowhead nor beluga whale migration times changed significantly in spring, but bowhead whales migrated significantly later in fall from 2008–2018. There were no clear relationships between bowhead whales and the environmental variables, suggesting that the DBO 6 region is a migratory corridor, but not a feeding hotspot, for this species. Surprisingly, beluga whale acoustic presence was related to zooplankton biomass near the mooring, but this is unlikely to be a direct relationship: there are likely interactions of environmental drivers that result in higher occurrence of both modeled zooplankton and belugas in the DBO 6 region. The environmental triggers that drive the migratory phenology of the two Arctic endemic cetacean species likely extend from Bering Sea transport of heat, nutrients and plankton through the Chukchi and into the Beaufort Sea.

## Introduction

The Arctic, particularly the Pacific Arctic, is where climate change is having the most dramatic effect on the environment [1]. First in 2007 and then in 2012, sea ice extent in the Arctic reached historic and dramatic lows [2]. Including these two extreme years, decreases are most pronounced in fall months where the open water season has been extended by 6 to 11 days per decade [3,4]. Such extreme changes in the physical environment should lead to changes across the ecosystem; the Distributed Biological Observatory (DBO) is framework that has been proposed as a “change detection array” to document these changes through repeated measurements at fixed locations over long-time scales [5,6].

Marine mammals have been considered sentinels of change [7,8], and in the Arctic, this is particularly true of ice-obligate species [the ice seals, walrus (*Odobenus rosmarus)* and polar bears (*Ursus maritimus*)] because loss of sea ice is loss of habitat that is used as a platform for resting, pupping, and hunting. How sea-ice reduction will affect Arctic cetaceans is less clear. Of the Arctic cetaceans, only bowhead (*Balaena mysticetus*) and beluga (*Delphinapterus leucas*) whales occupy the Pacific Arctic annually and migrate northwards in spring from the Bering Sea into the Chukchi and Beaufort Seas to feed.

Bowhead whales begin to migrate northwards in April from wintering grounds in the Bering Sea prior to sea ice breakup. Why bowhead whales begin their migration prior to the spring bloom is unknown but there is speculation that migration begins when copepods on the Bering Sea floor come out of diapause and are no longer aggregated on the seafloor [9]. Bowhead whales spend much of the summer in the Canadian Beaufort feeding on dense swarms of copepods before migrating westward towards the Chukotka coast of Russia and then southwards through the Bering Strait in late fall [10]. Bowhead whales are thought to feed primarily upon copepods in their summer feeding grounds in Amundsen Gulf and adjacent shelf waters of the eastern Beaufort Sea [11]. It has been hypothesized that the autumn migration from Canada begins when the copepods migrate too deeply for upwelling to lift them onto the shelf [11]. As bowhead whales migrate west during the fall migration, euphausiids (krill) from the Bering and Chukchi seas become more commonly consumed [12,13].

Two populations of beluga whales occupy the Beaufort Sea in summer and fall, the Eastern Chukchi Sea (ECS) and the Eastern Beaufort Sea (BS) populations. BS belugas migrate northwards with bowhead whales in spring traversing areas with very high ice concentrations and ECS belugas follow a month or so later [14]. In fall, BS belugas migrate west in August while ECS belugas migrate later, possible due to increasing sea ice concentrations or due to shifts in food resources [15,16]. While animals from these two populations overlap in the Beaufort Slope region in September, it is the ECS animals whose summer and fall home range encompasses the western Beaufort Sea slope [14]. The Barrow Canyon region and the shelf break east from there are considered high use areas for beluga whales [14,17]. Beluga whales in the Beaufort Sea are thought to consume Arctic cod (*Boreogadus saida*) but stomach contents from harvested belugas indicate that they consume a great variety of species include crustaceans, cephalopods and other fish [18,19].

In the Barrow Canyon region, both bowhead and beluga whales seem to take advantage of wind conditions that intensify the Beaufort shelfbreak jet, the continuation of the Alaskan Coastal Current (ACC) that flows eastward along the edge of the Alaskan Beaufort Sea shelf [20,21]. When the ACC and associated front are well defined, beluga whales are hypothesized to feed on prey accumulated by this front [22] but when easterly winds are strong, and disrupt the ACC, this species appears to forage at the Atlantic water (AW) boundary in Barrow Canyon [23]. Similarly, strong easterly winds promote upwelling, including of zooplankton, onto the Beaufort Sea shelf [24]. A subsequent relaxation of these winds and re-establishment of the ACC “traps” euphausiids on the shelf where they provide food for migrating bowhead whales [24–26]. Although likely influenced by similar environmental processes, these two cetacean species occupy adjacent habitat with bowheads more common on the Beaufort shelf and belugas at the shelf break and further offshore [14,23,27,28].

Based on high productivity, biodiversity and rapid ecosystem changes, the Barrow Canyon area has been designated as one of the Distributed Biological Observatory (DBO) regions (Region 5, [5,29]). The DBO began as a series of five regional “hotspot” stations along transect lines in the northern Bering and the Chukchi Sea. The overarching goal of the DBO is to detect environmental changes in the Pacific Arctic by regular shipboard monitoring of biophysical responses in each of the 5 DBO regions [6,29]. In 2015, the DBO expanded into the Beaufort Sea with the addition of three regions that include an oceanographically well-monitored location at ~152W along the Beaufort shelf break (Region 6) as well as regions in the central Beaufort Sea (Region 7) and the Cape Bathurst polynya (Region 8).

Lin et al. [30] examined the climatological monthly means of bowhead and beluga whale acoustic detections in Region 6 from 2008–2012 and related acoustic detections in spring to the northward migration of both species. In that study, the fall westward migration of both species was coincident with increased shelf break upwelling intensity in October [30]. Here we test the influence of environmental drivers on bowhead and beluga whale weekly, seasonal and interannual acoustic occurrence from 2008–2018, and examine changes in spring and fall migration timing in relation to sea ice melt and freeze-up dates.

## Methods

**Fig 1:**
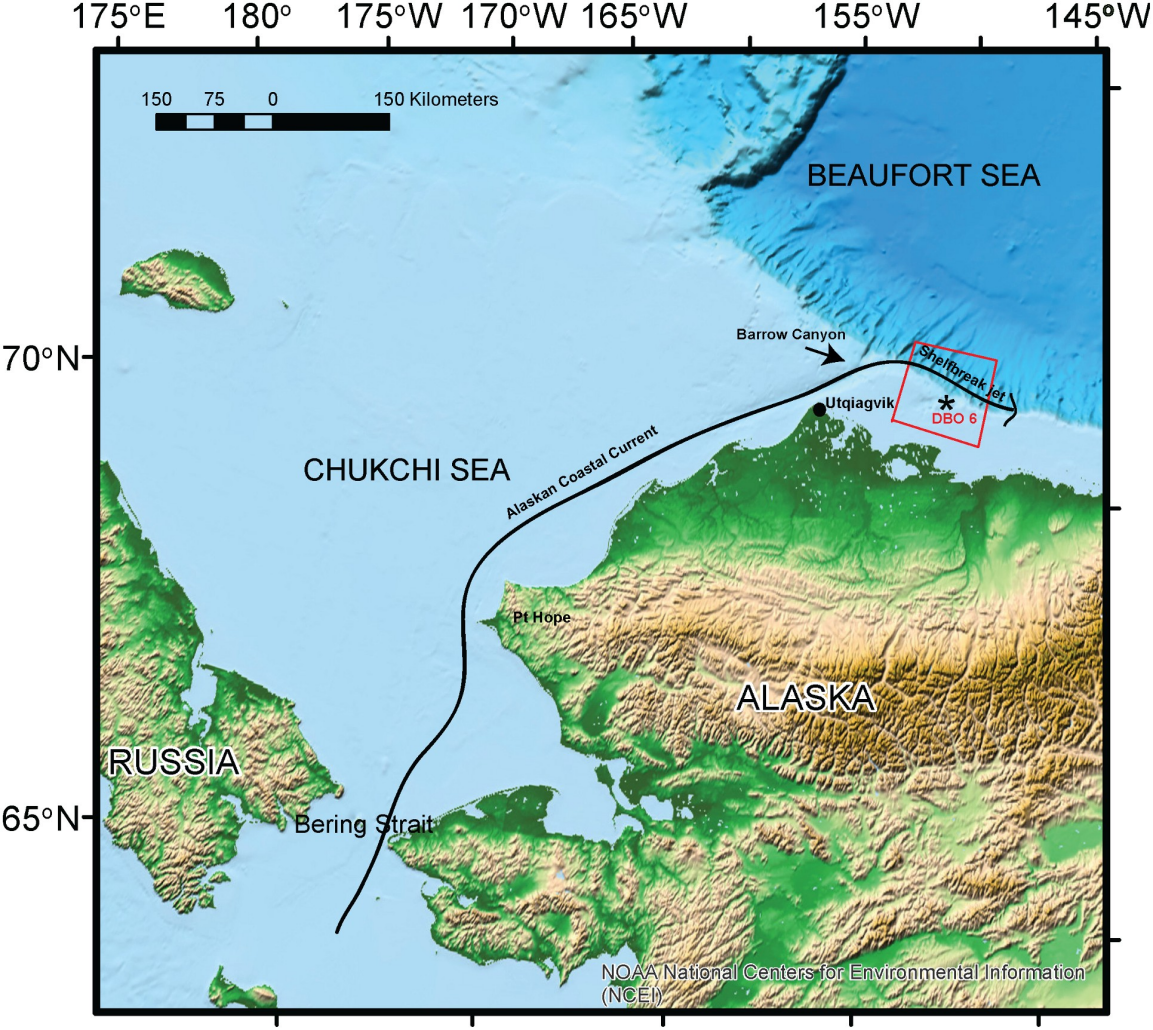
Map of study area. DBO 6 boundaries shown as red box, the mooring location as an asterix and the Alaskan Coastal Current and its extension, the Beaufort shelfbreak jet shown as a black line.

Passive acoustic data were collected from an Aural-M2 instrument package deployed annually from September 2008-July 2014 and September 2016-October 2018 at ~71.4°N, 152.0°W (Fig 1, Table 1). Instrument details including locations, depths, duty cycle and sample rates are given in Table 1. Data were archived onboard the instrument. Upon retrieval of the instruments, 60 s long spectrograms from 0.01–4 kHz (frame size 2048 samples, 50% overlap, Hann window) of each acoustic data file were visually inspected in Ishmael3.0 [31] for the presence of at least one beluga and/or bowhead whale signal per file. Faint signals, or those that were masked by noise, were verified aurally when needed.

**Table 1:** Annual deployment details for the hydrophone moored in DBO 6.

Year	Location	Start date	End date	Depth (m)	Sample rate (Hz)	Duty cycle (min/min)
2008–2009	71.46 N 152.25 W	16 Aug 2008	15 Aug 2009	134	8192	9/30
2009–2010	71.45 N 152.51 W	17 Aug 2009	12 Aug 2010	90	8192	9/30
2010–2011	71.41 N 152.00 W	25 Sept 2010	30 Aug 2011	163	16384	15/60
2011–2012	71.41 N 152.01 W	1 Sep 2011	28 Aug 2012	161	8192	10/30
2012–2013	71.41 N 152.00 W	31 Aug 2012	1 Sep 2013	166	8192	10/30
2013–2014	71.41 N 152.00 W	7 Sep 2013	18 Jul 2014	168	8192	10/30
2016–2018	71.39 N 152.05 W	13 Sep 2016	30 Oct 2018	147	16384	5/60

Beginning in 2016 the mooring was deployed for two years before recovery, so the duty cycle was decreased to ensure year-round acoustic coverage.

**Fig 2:**
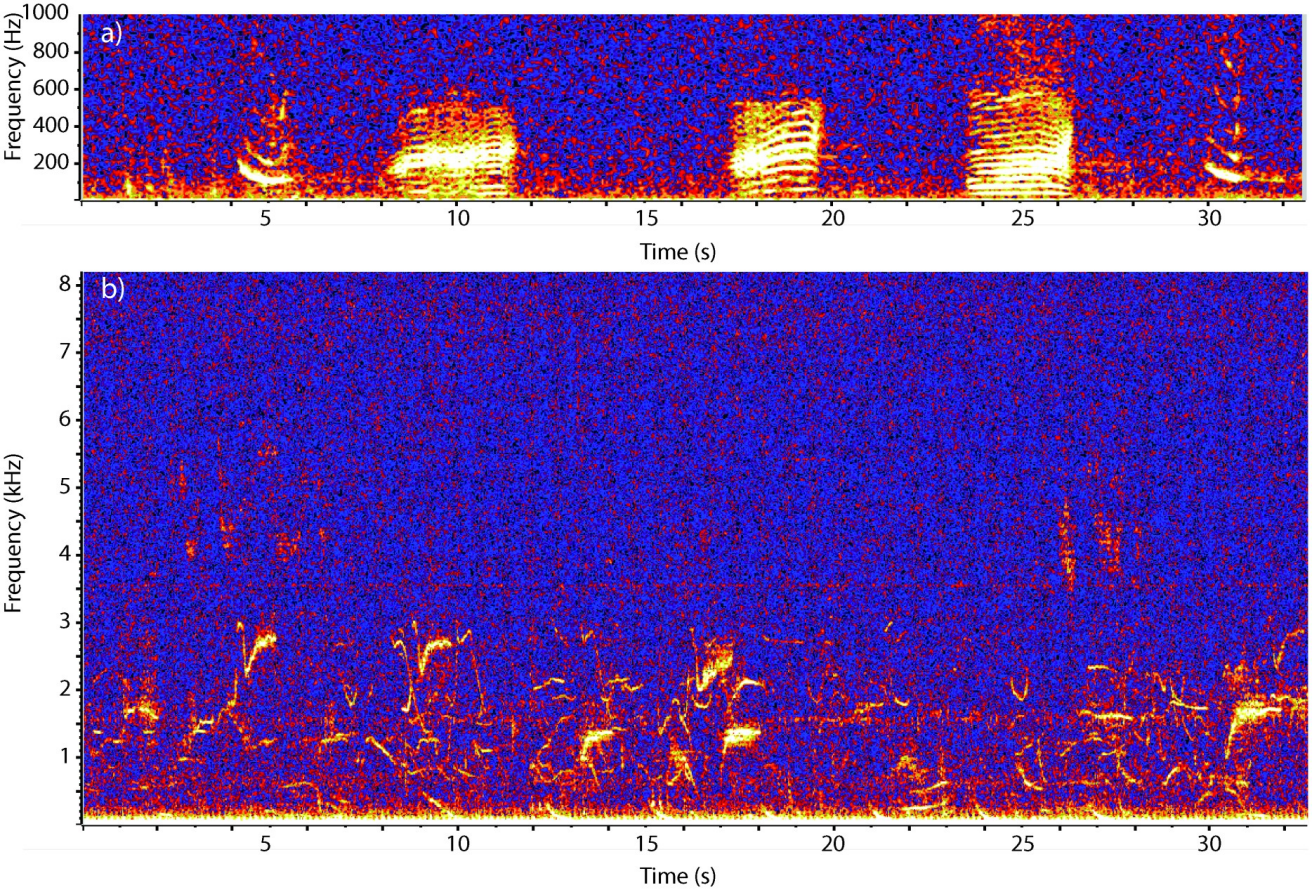
Spectrograms of characteristic bowhead and beluga whale signals. a) Five bowhead whale calls recorded on 11 July 2017 (FFT 2048, 50% overlap, Hann window). b) Beluga whale calls recorded on 4 May 2017 (FFT 1024, 50% overlap, Hann window). Note the different frequency axes for the two species’ signals.

Bowhead and beluga sounds are readily distinguishable from each other and from other Arctic species (Fig 2). Bowhead whale calls are low-frequency signals (usually between 30–500 Hz), that may be frequency- or amplitude-modulated and last from 0.5 to 5 s long [32,33]. Beluga whale calls are higher frequency (400 Hz-20 kHz), and shorter (0.1–1 s long) and for BS belugas, have peak frequencies under 4 kHz [34]. Based on this, although the bandwidth of our data did not cover the highest frequency ranges of beluga whale calls, we do not believe that we missed tonal signals with our lower sample rate. In general, bowhead whale calls can be detected out to 30 km while beluga calls can only be detected to about 3 km [35,36]. The overall annual weekly mean for each species was determined by averaging the number of hours per day by week with vocalizations from all available data from 2008–2018.

To determine if the shorter duty cycle in 2016–2018 (see Table 1) resulted in fewer detections during these years than in previous years we performed a t-test assuming unequal variances to test whether the overall mean number of hours per day with calls for the months of May to October differed significantly between 2008–2014 and 2016–2018. They did not, for either bowhead (p = 0.62) or beluga (p = 0.37) whales, therefore we proceeded with all further analyses under the assumption that duty cycle would not impact our results.

To determine if whales might be passing the mooring location earlier in the spring or later in the fall over the decade from 2008–2018, following Hauser et al. [16] we determined the cumulative distribution of days with bowhead or beluga vocalizations each year and took the 95% quantile of the cumulative distribution of day of year (DOY) for acoustic detections from 2008–2018 after 30 September for fall migration and after 15 March for the spring migration. To determine if passage date was related to sea ice area, we determined the DOY for melt and freeze-up using the method of Laidre et al. [3] to determine transition thresholds in spring and fall. We used 5 day trailing average daily sea ice area (km^2^) for the Beaufort Sea from the National Snow and Ice Data Center (NSIDC). The daily sea ice index products (25 km x 25 km resolution) are derived from the *Near-Real-Time DMSP SSM/I-SSMIS Daily Polar Gridded Sea Ice Concentrations* and *Sea Ice Concentrations from Nimbus-7 SMMR and DMSP SSM/I-SSMIS Passive Microwave Data* [37]. Simple linear regression was used to test the hypothesis that whale acoustic departure from the area was not related to DOY or transition date.

We examined whether environmental drivers (water temperature in the upper 10 m, sea ice, and modeled zooplankton biomass) that appear to drive the occurrence of bowhead and beluga whales near Barrow Canyon [22–26] function in a similar manner in DBO region 6. The Biology-Ice-Ocean Modeling and Assimilation System (BIOMAS), [38–40] was used to estimate the mean weekly biomass of copepods and predatory zooplankton (in mmol-N/m^3^), sea ice concentration, and ocean temperature (°C), in a 1° by 1° region around the mooring location for the months of July, August, September, and October. These are the months during which both cetacean species are known to be actively feeding in the Beaufort Sea and the months for which there is detectable modeled biomass.

We used linear mixed effects models to relate bowhead and beluga detections to our environmental covariates [41]. Bowhead and beluga whales were modeled separately, and the response variable consisted of the average number of hours per day (within each week, July–October) with detections. Year was modeled as a random effect. Residuals were autocorrelated in time, so we also included a first-order autocorrelation parameter (i.e., AR(1)) that was indexed by week within years. All models were fit using package *nlme* in R [42]. We ranked models using AICc [43] and maximum likelihood; we then used restricted maximum likelihood to estimate parameters from the best approximating model, because it provides the best estimates of parameter error [41].

## Results

### Bowhead whales

**Fig 3:**
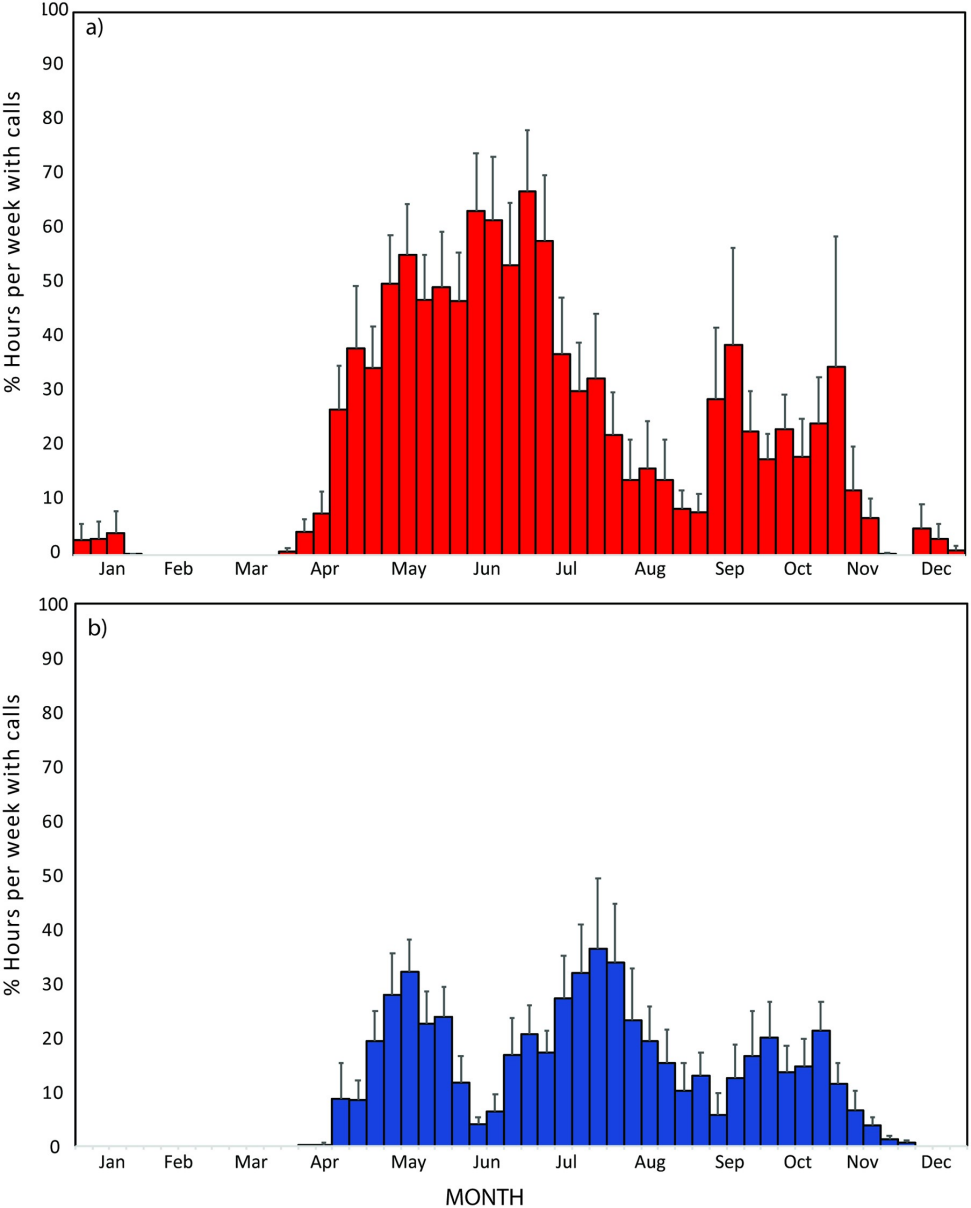
Decadal mean (+ S.E.) number of hours per week from 2008–2018. a) bowhead and b) beluga whale calls recorded on a hydrophone moored in DBO region 6.

**Fig 4:**
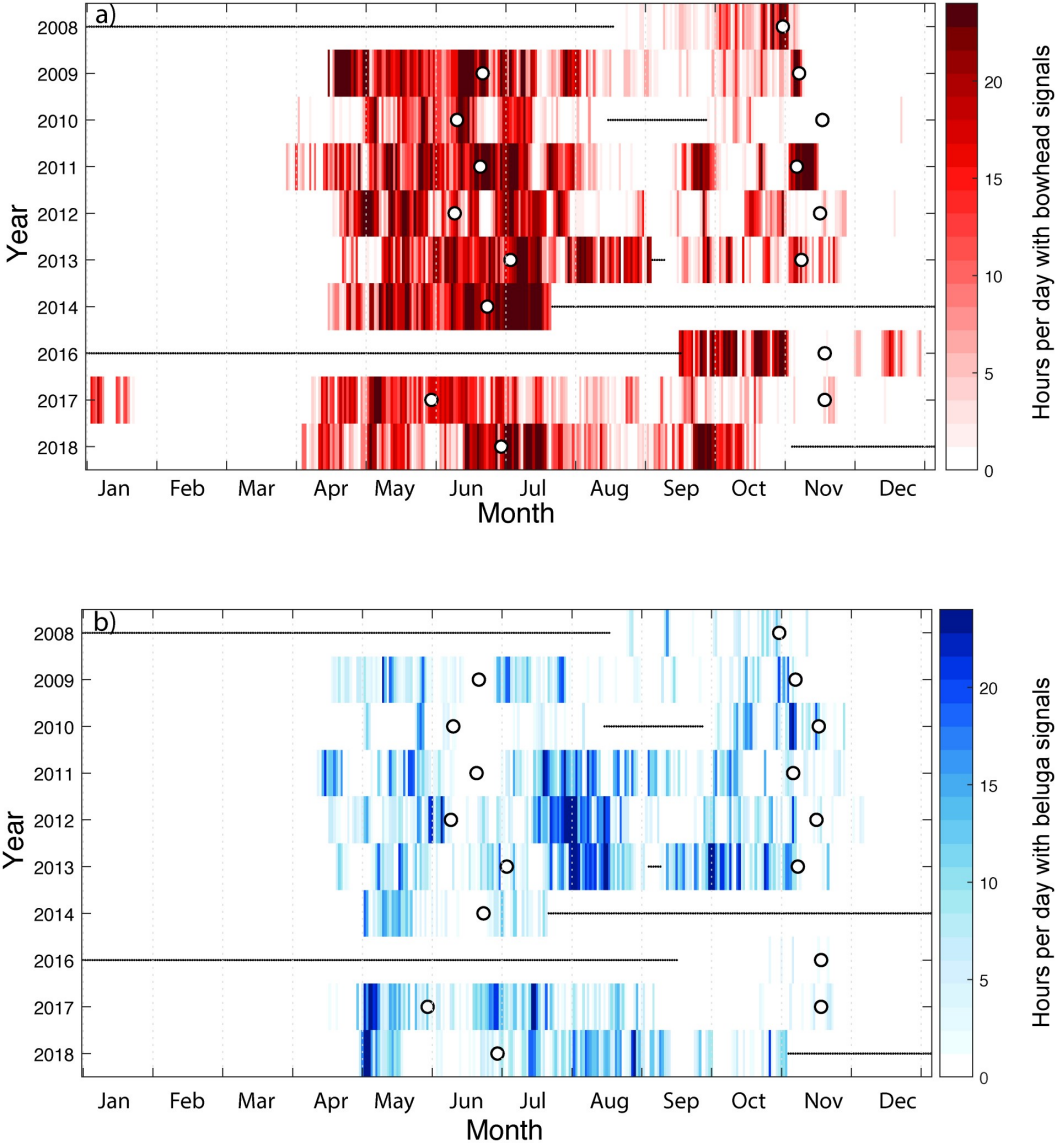
Heatmaps of hours per day with acoustic detections by year from 2008–2018. a) bowhead whales and b) beluga whales. Timing of melt and freeze up transitions by year are shown as white circles. Black horizontal lines indicate missing data. Note that there were no data for the entirety of 2015 so that year is not shown.

Bowhead whale calls were detected starting in early-April through late November. The overall annual pattern of call detections was bi-modal with a broader peak from May through July and a second, distinct but smaller, peak from mid-September to mid-November (Fig 3a). Beginning in mid-April bowhead whale signals were detected daily and in multiple hours per day until early July after which time detections became less regular and occurred in “bouts” of several days or hours with detections followed by fewer or no detections. During spring, bowhead whales began to be heard when sea ice concentrations were high, long before the spring melt transition date. In contrast, fall acoustic detections usually ceased not long after the freeze-up transition date (Fig 4a). There were years, in particular 2016 and 2017, but also 2010 and 2012, when bowhead calls were recorded into December. In the late fall and winter of 2016–2017 bowhead whales were in the region into January, suggesting that they were only absent from the Beaufort Sea for 2 months before returning in April.

**Fig 5:**
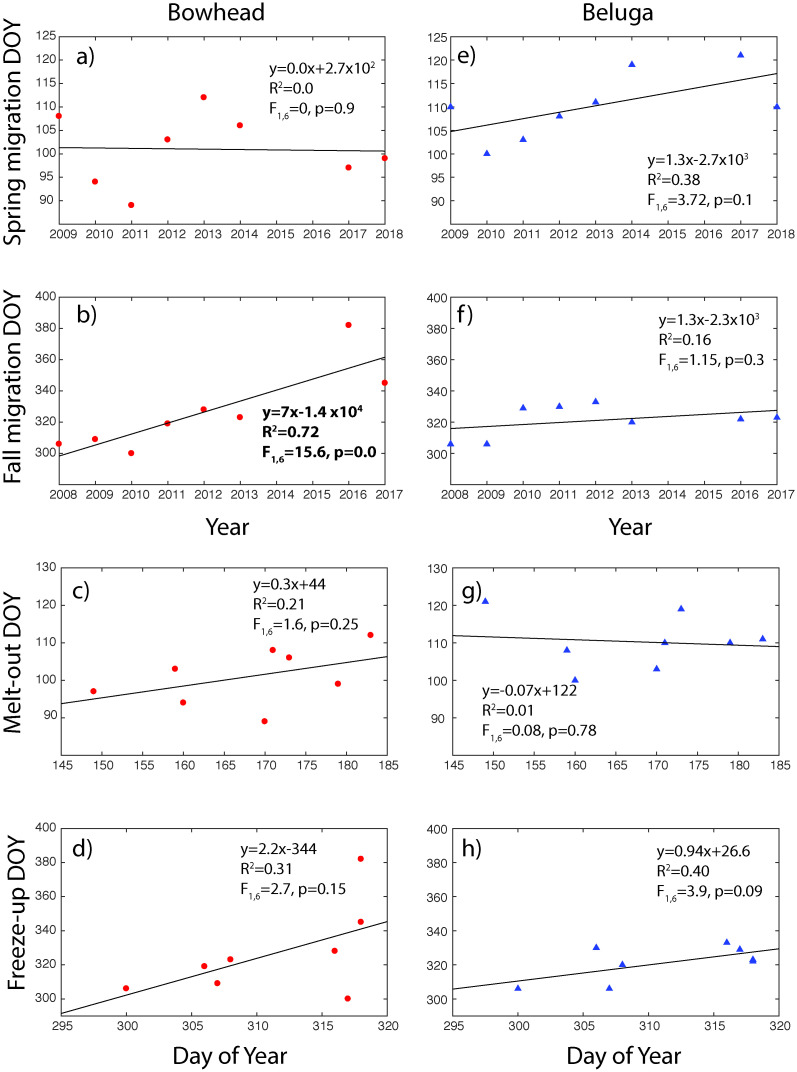
Linear relationship of acoustically detected migration passage day of year (DOY) by year and by melt-out and freeze-up DOY from 2008–2018. a) bowhead whale migration DOY in spring; b) bowhead whale migration DOY in fall. DOY over 365 indicates passage date in January 2017; c) bowhead whale migration and spring melt-out DOY; d) bowhead whale migration and fall freeze up DOY; e) beluga whale migration DOY in spring; f) beluga whale migration DOY in fall; g) beluga whale migration and spring melt out DOY; h) beluga whale migration and fall freeze up DOY. Trend, fit (r^2^), and F-statistics of acoustic migration DOY by year and sea ice thaw/freeze up DOY over time are shown on each plot. Significant statistical relationships (*p*<0.05) are shown in bold type on the figures.

The 95% quantile date of bowhead whale spring migration past the hydrophone showed no clear annual trend from 2009–2018 (r^2^ = 0.0, P = 0.9, Fig 5a). Bowhead whales appeared to migrate earlier when the spring melt-out date was earlier although this was not significant (p = 0.25, Fig 5c). The 95% quantile date of fall migration occurred 7 days per year later from 2008–2018 (r^2^ = 0.72, P = 0.0, Fig 5b). When compared to date of annual freeze-up, this occurred 2.1 days later per year but was not statistically significant (r^2^ = 0.31. P = 0.15, Fig 5d). Data from 2008–2013 showed that migration occurred only 4.6 days later per year. The greater increase, after including 2016–2018 data, arose from the winter of 2016–17 during which time bowhead whales stayed in the Beaufort Sea into mid-January before migrating south.

There were no significant relationships between monthly mean bowhead call occurrence, month, ocean temperature or modelled zooplankton biomass. The model with the lowest AICc suggested that calls varied by month, largely because call rates declined in November. Temperature was also included in this model, but the relationship was not statistically significant (*p* = 0.14). The next best approximating model, which received virtually equal weight (Δ AICc 0.15), included only month.

### Beluga whales

Beluga whales were detected from mid-April to mid-November annually from 2008–2018. The seasonal mean showed three peaks in detection: two in the spring and a third in the fall (Fig 3b). The first detection peak occurred from mid-April until early June and was present up to two full months before spring melt transition date (Fig 4b). These signals are assumed to be produced by the BS population of beluga whales [14]. The second detection peak, from late June through mid-August, occurred as sea ice declined and then disappeared from the study region (Fig 3b). These signals are hypothesized to come from the ECS population of beluga whales which migrate after the BS belugas and then remain resident in the Barrow Canyon/eastern Beaufort Sea shelf area during the summer. The final acoustic mode occurs in the fall from mid-September to mid-November and may represent individuals from both populations during their fall migration back towards the Bering Sea (Fig 3b).

Although there was some inter-annual variation in the timing of acoustic detections in both spring and fall, there were no significant differences in the dates when belugas first and last passed the hydrophone location (Fig 5). The 95% quantile date of beluga whale spring migration past the hydrophone occurred 1.3 days later annually from 2009–2018 but the trend was not significant (r^2^ = 0.38, P = 0.1, Fig 4e). There were no changes in beluga spring migration with melt-out date (Fig 5g). The 95% quantile date for beluga whale fall migration suggested only a 1.3 day per year delay in migration but this trend was not significantly different from 0 (r^2^ = 0.16, P = 0.3, Fig 5f). There was a delay of 0.9 days per year in fall migration when compared to freeze-up date, but again, it was not statistically significant (r^2^ = 0.4, P = 0.09, Fig 5h).

The best approximating model for weekly beluga call occurrence included zooplankton density from the BIOMAS model and no other covariates. This model suggested that the average number of hours per day with beluga calls increased from an average of 0.4 hr (*SE* = 1.0) when zooplankton density was 0.3 mmol-N/m^3^ to 8.0 hr (*SE* = 1.0) when zooplankton density was 1.0 mmol-N/m^3^.

## Discussion

Both bowhead and beluga whales were acoustically detected at DBO 6 from 2008–2018 with clear seasonal patterns in detections that likely reflect the migratory movements of the three populations (one bowhead and two beluga) known to inhabit the Pacific Arctic. In all years, both bowhead and beluga whales migrated north and eastwards in the spring well before sea ice break-up. In the fall, however, the decline in acoustic detections of both species coincided with the date of sea ice freeze-up. The southward movement of bowhead whales in the Bering Sea from the Chukotka coast in fall has been correlated with sea ice formation based on whales instrumented with satellite tags [9]. Both species were acoustically detected almost every week from the spring through fall in the DBO6 region (Fig 3) although examination of the daily occurrence suggests that acoustic detections can vary by day and the number of hours per day (Fig 4). Further, for both species there is a clear lull from mid-August to mid-September at this site.

The decade from 2009–2018 has had the ten lowest minimum sea ice extents in the satellite record, which began in 1979 [37]. In the Beaufort Sea spring break up occurred 21 days earlier in 2002–2011 than it did from 1982–1991 and fall sea ice formation occurred 2 weeks later [3]. That trend has continued. As a result, the number of open water days has increased annually in bowhead whale core use areas to the east and west of DBO 6 [44]. Chukchi beluga whales have been found to delay migration out of the Beaufort and Chukchi Seas by 2–4 weeks in the fall over the past two decades although BS belugas showed no evidence of such phenological changes [16]. At DBO 6, the DOY for the onset of the spring migration did not change significantly for bowhead or beluga whales. In contrast, in the fall, the DOY for bowhead whale passage showed a significant change, increasing by 7 days per year. The DOY for belugas passing the mooring also occurred later over time, but this change was not significant when examining data by year. The change was significant when compared to the freeze-up DOY [16] until data from 2016–2018 were added.

There are only three species of truly Arctic whale, one baleen whale, the bowhead, and two toothed whales, belugas and narwhals (*Monodon monoceros*). Of the three species, narwhals are perhaps the most vulnerable to rapid changes in habitat because they are highly specialized to feed on relatively few species of fish (primarily Greenland halibut, *Reinhardtius hippoglossoides*), over a limited area and season. Later fall freeze up has been suggested as a reason for recent increases in narwhal entrapments in ice, presumably because the animals delayed their departure from summering grounds [45,46]. In a similar way, beluga whale distribution has expanded from nearshore into open waters as sea ice extent has decreased in both spring and fall [47,48]. Unlike narwhals, however, the number of documented ice entrapments for this species have decreased [47]. Also, in contrast to narwhals, beluga whales have a large population size, greater geographic range and much broader diet that ranges from invertebrates to fish [18,19,49].

Numbers of bowhead whales are increasing in the two populations for which there are reliable data [50,51]. Further, the body condition of western Arctic bowhead whales appears to be improving and this change is related to the decrease in summer sea ice that may result in increased upwelling and prey production [52]. In regions that are considered foraging “hot spots” for bowhead whales, and which overlap hotspots for beluga whales, the number of open water days in the western Beaufort Sea slope and shelf region have increased by up to 25 per decade from 1979 to 2014 [44].

Based on aerial survey data from 1979–1983, the bowhead whale spring migration past Point Barrow began in late April or early May [53] but more recently bowhead whales have been arriving earlier at Utqiaġvik in the spring [44]. In the 1980s and 1990s, when seasonal sea ice was considerably older, thicker and more extensive for most of the year, bowhead whales migrated out of the Beaufort Sea from late August through October and into the Bering Sea by November [54]. Now, though, bowhead whales are remaining in the Beaufort Sea longer, as evidenced by recent data from the Canadian Beaufort where bowhead whales were detected in late December in 2016 [55], the same year in which they were recorded in our data, just further west, into January 2017. More extreme evidence of this delayed migration includes mid-winter sightings and acoustic detections of bowhead whales in the eastern Canadian Beaufort in 2018–2019 [56]. While the seasonal residency of bowheads in the Beaufort seems to be extending with declining sea ice [55–57], particularly in the fall, there is less evidence that they are changing their overall distribution. As in the 1980s to early 1990s [28], bowhead whales are still found predominately on the Beaufort Sea shelf suggesting that distance to the coast, and/or bathymetry is a more important driver of occurrence than distance to sea ice [44,58]. However, distance to shore is not always the dominant driver of bowhead distribution in autumn; in the fall of 2019, which is outside the range of data presented here, few bowhead whales were seen by aerial survey observer in September and October and sighted whales were farther offshore than they have been historically [28] and Native hunters did not find whales in the nearshore Beaufort or Chukchi Sea [59].

There were no clear associations between bowhead vocalization occurrence at the mooring location and environmental drivers, beyond the seasonality of each. This is perhaps to be expected, as this area largely serves as a migratory corridor linking summer feeding grounds in the Canadian Beaufort with fall feeding grounds at Utqiaġvik and in the Chukchi Sea. Although feeding is well-documented in the Alaskan Beaufort Sea [e.g., 60,61] feeding events tend to be less consistent and of shorter duration than what is observed within core use areas [e.g., 62,63].

The majority of zooplankton that enter the western Beaufort originate in the Bering Sea and are advected northwards through Bering Strait and into the Chukchi and western Beaufort seas [24,64]. Zooplankton are advected on currents that typically run eastwards along the shelf break [24,25]. There are no known, consistently used, bowhead core-use areas between the Tuktoyaktuk and Herschel Island in the east (i.e., eastern Beaufort) and Point Barrow in the western Beaufort [9]. Both those regions have mechanisms that consistently trap and aggregate zooplankton which are lacking in the DBO6 area. Although bowhead whales are known to sometimes stop and feed on the shelf prior to reaching Point Barrow (i.e., during migration), those events are ephemeral and typically very short (i.e., on the order of a hours to a couple of days). Feeding events are sometimes associated with elevated discharge from rivers which create freshwater fronts that aggregate zooplankton [e.g., 60]. Unfortunately, climatological (long-term mean) freshwater inputs from river systems are used in the BIOMAS model [28]. Hence, even if an event that aggregated zooplankton occurred, it is unlikely the BIOMAS model would pick it up. As such, it is no surprise to us that the modeled zooplankton do not explain the rate of bowhead calling.

Beluga whales in the Beaufort Sea represent two different populations with different migratory timing in both the spring and fall [14]. Both aerial survey and satellite telemetry data illustrate beluga whale preference for off-shelf waters [14,15,22,65]. Like bowhead whales, bathymetry, rather than ice, appears to be the strongest predictor of beluga whale habitat in the summer and late fall [66]. This may be why, while there is a trend towards later migration in the fall in the beluga whale data, it was not significant. The most obvious signal in the acoustic data is the trimodal distribution of beluga calls representing the spring migrations first of BS belugas in April and May followed by ECS belugas which arrive in June and remain in the western Beaufort Sea until August [14]. The third pulse seen in Fig 2b is likely representative of animals in both populations on their way west towards Bering Strait [15]. The overall reduced occurrence of beluga whale sounds as compared to bowheads is very likely due to the reduced range over which the higher frequency beluga whale signals can be detected versus low-frequency bowhead signals.

In contrast to the bowhead whale results, zooplankton densities from the BIOMAS model were a statistically significant predictor of beluga vocalizations. We suggest that the occurrence of belugas near the mooring has little to do with zooplankton density; rather, it is likely that zooplankton density from the BIOMAS model is correlated with something else that is linked to beluga density. To better understand this, we need to decompose the BIOMAS model to determine what exactly is driving rates of modeled zooplankton.

Prior studies have shown that belugas are more likely to use Barrow Canyon when winds promote a well-defined ACC, providing a front for belugas to forage along [16]. We suspect that wind patterns that strengthen the ACC, also strengthen the shelfbreak jet [21], which likely also delivers more zooplankton to the mooring region within the BIOMAS model. Hence, zooplankton density from the BIOMAS model may be a proxy for the strength of the front associated with the shelfbreak jet. The relationship between winds and beluga distribution is likely complex, requiring the integration of wind direction and speed over periods longer than a day and potentially lagged in time. Such an analysis is clearly warranted but is outside the scope of this manuscript. Regardless, that beluga calls are related to a covariate from the BIOMAS model suggests that the mooring site is more than a migratory corridor for beluga whales, which is supported by satellite tagging studies [e.g., 14].

That beluga, and not bowhead whale, calls might be related to a lower trophic ecological covariate was somewhat surprising. The prey fields for both belugas (arctic cod and other species) and bowheads (zooplankton) in Alaskan and Canadian regions of the Beaufort Sea fundamentally depend on regional upwelling winds [24,26,67] the variability of which are largely attributable to the strength and location of the Beaufort Sea high pressure cell [68,69]. However, secondary mechanisms that establish strong fronts (convergence zones) are necessary to aggregate prey to enable belugas and bowheads to forage in an energetically efficient manner. These secondary mechanisms only occur with some regularity in the coastal Beaufort Sea near Amundsen Gulf/Cape Bathurst, Canada [9,11,69] and Utqiaġvik, Alaska [9,22,24–26]. As noted above, between these two core use areas, aggregations of prey on the central Beaufort shelf occur much less frequently because they require anomalously high river discharges to occur contemporaneously with enhanced tidal mixing to establish frontal zones capable of concentrating prey [60].

The environmental triggers that dictate the migratory phenology of these two Arctic endemic cetacean species likely extend from the Bering Sea through the Chukchi Sea and into the Beaufort Sea. To begin to understand this, future research directions include examining the fine-scale timing of species’ movements across the DBOs, in situ measurement of prey fields, and how near- and far-field wind forcing influences the currents that drive transport of heat, nutrients, and plankton into the Beaufort Sea, ultimately dictate timing when and where Arctic cetaceans occur.

## Supporting information

S1 Fig (TIF)

S2 Fig (TIF)

S3 Fig (TIF)

S1 Table Bowhead whale acoustic detections from 2008–2018. Mean number of hourly files per day with at least one bowhead whale acoustic detection. NaN indicates missing data.
(XLSX)

S2 Table Beluga whale acoustic detections from 2008–2018. Mean number of hourly files per day with at least one beluga whale acoustic detection. NaN indicates missing data.
(XLSX)

S3 Table BIOMAS output for 71N 152.3W. Daily values for biomass of copepods and predatory zooplankton (in mmol-N/m^3^), sea ice concentration, and ocean temperature (°C), in a 1° by 1° region centered on 71° N, 152.3°W.
(XLSX)
