# Supplementary figures and images for: Bowhead and beluga whale acoustic detections in the western Beaufort Sea 2008–2018
Source: PLoS One. 2021 Jun 28;16(6):e0253929. doi: 10.1371/journal.pone.0253929 (PMC8238202; doi:10.1371/journal.pone.0253929)

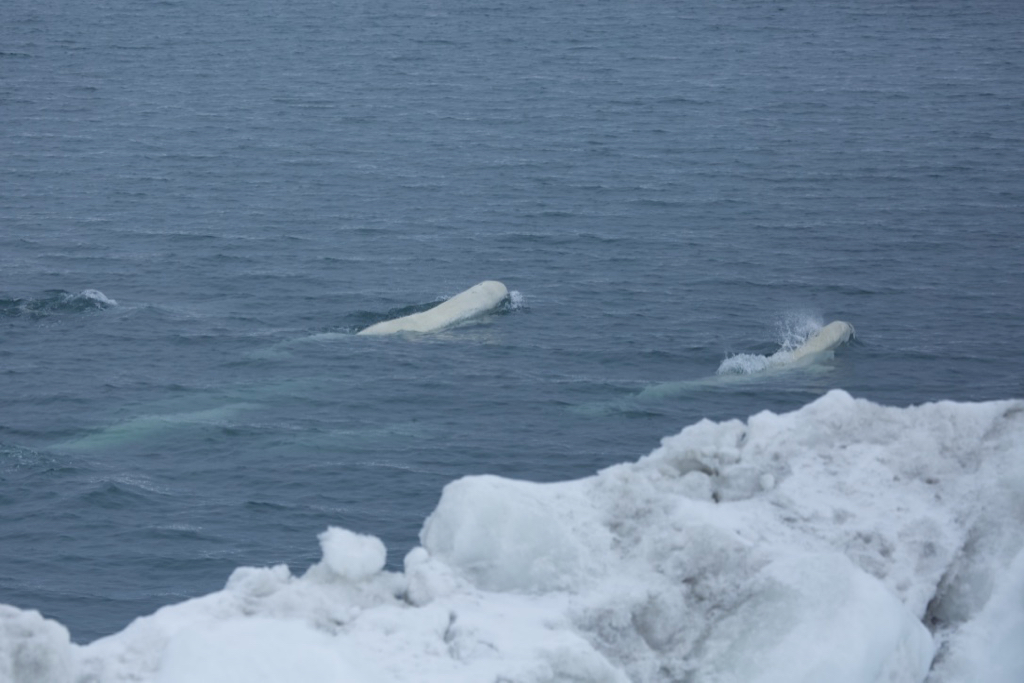

Supplement: S1 Fig — (TIF) [file pone.0253929.s001.tif]

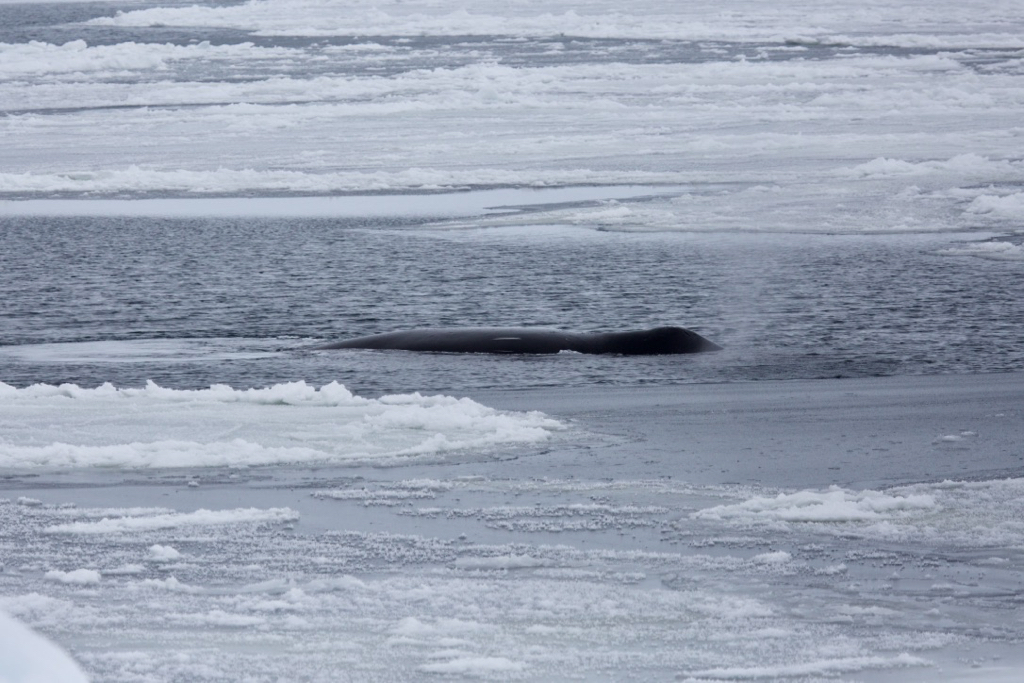

Supplement: S2 Fig — (TIF) [file pone.0253929.s002.tif]

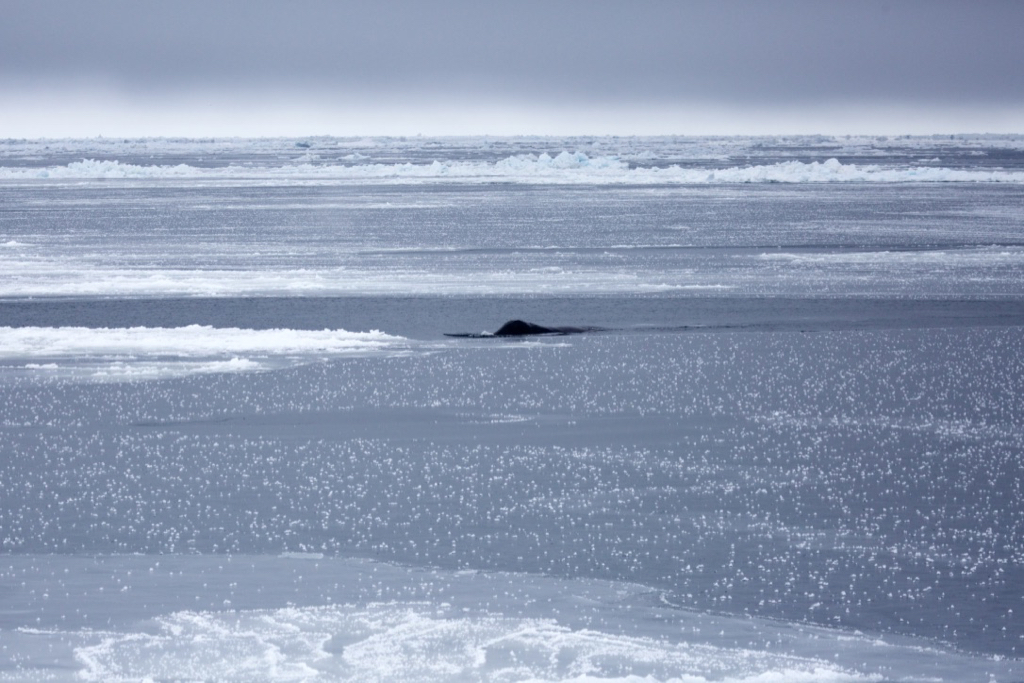

Supplement: S3 Fig — (TIF) [file pone.0253929.s003.tif]
